# Development and acceptability of PETS-Now, an electronic point-of-care tool to monitor treatment burden in patients with multiple chronic conditions: a multi-method study

**DOI:** 10.1186/s12875-024-02316-5

**Published:** 2024-03-01

**Authors:** David T. Eton, Kathleen J. Yost, Jennifer L. Ridgeway, Bayly Bucknell, Mike Wambua, Natalie C. Erbs, Summer V. Allen, Elizabeth A. Rogers, Roger T. Anderson, Mark Linzer

**Affiliations:** 1 Outcomes Research Branch, Healthcare Delivery Research Program, Division of Cancer Control and Population Sciences, National Cancer Institute, 9169 Medical Center Drive, Rockville, MD 20850 USA; 2 Department of Quantitative Health Sciences, Mayo Clinic, Rochester, MN USA; 3 Division of Health Care Delivery Research, Robert D. and Patricia E. Kern Center for the Science of Health Care Delivery, Mayo Clinic, Rochester, MN USA; 4 Hennepin Healthcare Research Institute, Minneapolis, MN USA; 5 Department of Family Medicine, Mayo Clinic, Rochester, MN USA; 6 Departments of Medicine and of Pediatrics, University of Minnesota, Minneapolis, MN USA; 7 Department of Public Health Sciences, University of Virginia, Charlottesville, VA USA; 8 Department of Medicine, Hennepin Healthcare and University of Minnesota, Minneapolis, MN USA

**Keywords:** Patient-reported outcomes, Self-management, Quality of health care, Primary health care, Multimorbidity, Quality of life, Patient-reported experience, Telehealth

## Abstract

**Background:**

The aim of this study was to develop a web-based tool for patients with multiple chronic conditions (MCC) to communicate concerns about treatment burden to their healthcare providers.

**Methods:**

Patients and providers from primary-care clinics participated. We conducted focus groups to identify content for a prototype clinical tool to screen for treatment burden by reviewing domains and items from a previously validated measure, the Patient Experience with Treatment and Self-management (PETS). Following review of the prototype, a quasi-experimental pilot study determined acceptability of using the tool in clinical practice. The study protocol was modified to accommodate limitations due to the Covid-19 pandemic.

**Results:**

Fifteen patients with MCC and 18 providers participated in focus groups to review existing PETS content. The pilot tool (named *PETS-Now*) consisted of eight domains (Living Healthy, Health Costs, Monitoring Health, Medicine, Personal Relationships, Getting Healthcare, Health Information, and Medical Equipment) with each domain represented by a checklist of potential concerns. Administrative burden was minimized by limiting patients to selection of one domain. To test acceptability, 17 primary-care providers first saw 92 patients under standard care (control) conditions followed by another 90 patients using the PETS-Now tool (intervention). Each treatment burden domain was selected at least once by patients in the intervention. No significant differences were observed in overall care quality between patients in the control and intervention conditions with mean care quality rated high in both groups (9.3 and 9.2, respectively, out of 10). There were no differences in provider impressions of patient encounters under the two conditions with providers reporting that patient concerns were addressed in 95% of the visits in both conditions. Most intervention group patients (94%) found that the PETS-Now was easy to use and helped focus the conversation with the provider on their biggest concern (98%). Most providers (81%) felt they had learned something new about the patient from the PETS-Now.

**Conclusion:**

The PETS-Now holds promise for quickly screening and monitoring treatment burden in people with MCC and may provide information for care planning. While acceptable to patients and clinicians, integration of information into the electronic medical record should be prioritized.

**Supplementary Information:**

The online version contains supplementary material available at 10.1186/s12875-024-02316-5.

## Background

Many chronic health conditions being managed by primary healthcare providers require continuous self-management on the part of patients. Self-management includes healthcare tasks and activities that must be routinely performed to remain healthy. This can include taking medications as directed, attending medical appointments, understanding and following medical advice, monitoring health, and maintaining diet and exercise regimens [1–3]. The personal work of healthcare can be burdensome to patients, especially when it interferes with the pursuit of other valued life activities [2]. This is captured in the concept of *treatment burden* defined as the workload of treatment and self-management for chronic conditions, its impact on patient functioning, and stressors that exacerbate it like healthcare financial concerns [1, 4]. Treatment burden is highly relevant to people with multiple chronic conditions (MCC) who are often challenged with integrating multiple, complex, and sometimes competing care regimens into daily life [2, 5]. Recently, research teams in North America and Europe have operationalized the concept of treatment burden in several patient, self-report measures [6–9]. However, to date there is no patient, self-report tool specifically designed to monitor and screen for treatment burden in clinic settings. In this report we describe the development and acceptability of implementing such a tool.

Routine assessment of treatment burden in the clinic has the potential to benefit both primary healthcare providers and the patients whom they serve. Lower patient-reported treatment burden is associated with better adherence to prescribed medical regimens, including medication, diet, and exercise [3, 8, 10]. Optimal patient adherence can lower the risk of exacerbation [11], promoting lower rates of hospitalization [11, 12], readmission [12, 13], and mortality [11, 12, 14]. Hence, attending to treatment burden during clinical consultations could lead to better long-term clinical outcomes. While providers are knowledgeable about many of the challenges faced by patients with MCC, they may be less aware of the severity of treatment burden and when patients may be considering lowering their adherence to prescribed care to cope with it [15, 16]. From the patient’s perspective, lower treatment burden is associated with higher self-efficacy [8, 17] and better well-being and quality of life [6, 7, 17, 18].

Emerging evidence supports that treatment burden is amenable to intervention. Lesage and colleagues [19] recently reviewed studies of medical interventions to reduce treatment burden in adults with long-term conditions. Of eleven intervention studies reviewed, 8 (73%) reported a significant positive impact of intervention on patient-reported treatment burden, most featuring a reduction in perceived workload. Studies were mostly small scale, focused on a single specific health condition (e.g., diabetes), and targeted simplification of a treatment regimen (e.g., changing a medication or medical device). One large, cluster-randomized trial conducted in the UK with multimorbid patients in primary care failed to demonstrate significant effects on treatment burden of a consensus-based, multi-disciplinary, patient-centered care approach compared with usual care (the 3D trial) [20]. However, compared with usual care, patients in the intervention group perceived higher care quality.

To date there are no treatment burden interventions that integrate patient-reported outcome measures (PROMs) as components of the intervention itself. Instead, the PROMs are used to assess intervention efficacy [19]. Yet there is growing support for the use of PROMs in clinical practice to support management of patients at the point of care [21, 22]. The Patient Experience with Treatment and Self-management (PETS) is a validated measure of patient-reported treatment burden [8, 17]. While both long-form (60 item) [17] and short-form (32 item) versions [23] are available, one application of PETS content that is needed is a rapid means of monitoring treatment burden at the point of care to inform patient-provider communication. Such information, if collected from patients and delivered to providers in an efficient and unobtrusive manner, has the potential to lead to changes in patient management that could lessen treatment burden. Therefore, the objectives of this study were to adapt the long-form (60 item) PETS to capture and briefly report a patient’s current treatment burden to healthcare providers at the point of care and to assess the acceptability of using such a tool in routine clinical practice.

## Methods

**Fig. 1:**
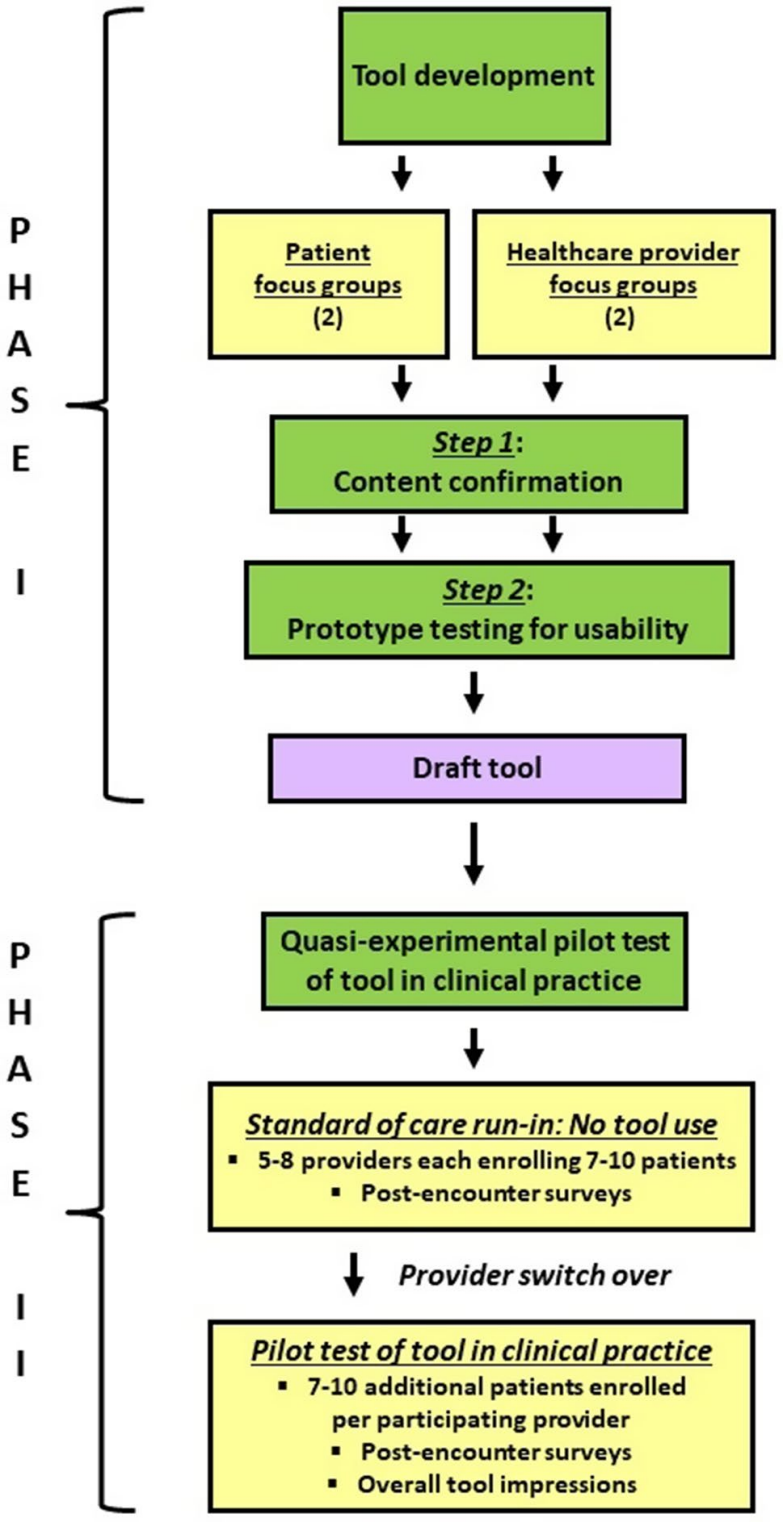
Study overview

The study proceeded in two phases (see Fig. 1). Phase 1 focused on tool development. Phase 2 assessed the acceptability of using the tool in clinical practice. Both phases were conducted at primary care-internal medicine clinics affiliated with the Mayo Clinic in Rochester, Minnesota (a small urban area) and Hennepin Healthcare in Minneapolis, Minnesota (a large urban area). Hennepin Healthcare houses Minnesota’s largest safety-net hospital that cares for many socially vulnerable and economically-disadvantaged patients. Institutional review boards at both medical centers approved of this research (Mayo IRB numbers: 16-010356 and 18-006429; Hennepin IRB numbers 17-4314 and 18-4625). All study procedures were conducted in accordance with relevant ethical guidelines and regulations.

### Phase 1: development

To adapt the PETS to a format that would enable efficient administration in a busy clinic setting, we engaged patient and provider stakeholders in separate focus groups to specify content from the 60-item version that should be included in a brief, web-based, point-of-care tool. With a focus to assess treatment burden in real time, we named the tool “PETS-Now.” The development phase had two steps: (1) content confirmation, and (2) usability testing. Patients were eligible to participate in the development if they were at least 21 years old, English speaking, had at least 2 primary care appointments at the affiliated clinic in the past 12 months, and had been diagnosed with 2 or more conditions from a pre-specified list of 20 chronic conditions often seen by primary care providers (e.g., diabetes, arthritis, COPD, hypertension, cancer). A recruitment letter was mailed to eligible patients, with instructions to contact the study coordinator by phone or email to be scheduled for the discussion group if interested in participating. The goal of content confirmation (Step 1) was to identify questions, either from the full 60-item PETS or from other topics offered by participants, that should be included in the PETS-Now tool. The purpose of usability testing (Step 2) was to obtain feedback on usability and acceptability of the PETS-Now prototype, specifically with respect to clarity of instructions, ease of navigation, clarity of PETS items, and time to complete.

We used methods similar to ones employed previously in developing and testing a web-based, point-of-care quality of life tool in diabetes [24]. We used a variety of recruitment methods including mail, phone, and in-person contact in clinic. Providers were eligible to participate if they provided primary care in the affiliated clinics. Providers from a wide range of clinical roles (e.g., physician, physician assistant, nurse, nurse practitioner, dietician, social work, pharmacist) were recruited by email at the Hennepin Healthcare site. At Mayo Clinic, we utilized time allocated for a regularly scheduled staff meeting to complete study activities. These meetings included physicians, physician assistants, and nurse practitioners. The original intention was to include the same provider and patient participants in both the content confirmation and prototype testing groups; however, we recruited additional individuals to attend the latter groups to ensure an adequate number of participants for robust discussion. Patients received $20 remuneration and a parking voucher for participating in a content confirmation focus group and $15 remuneration and a parking voucher for participating in usability testing. Providers were offered $100 remuneration for participating in content confirmation focus groups and $50 remuneration for prototype testing. Food and beverages were also provided in all focus groups.

Step 1 (content confirmation) focus groups were conducted between May 3, 2017 and June 21, 2017 by two members of the study team trained in qualitative research methods (KJY and JLR) using a semi-structured interview guide that explored participant experiences related to the research topic before seeking input and engaging participants in activities for the purpose of confirming content for PETS-Now, including existing content in the full PETS. Patient groups began with exploratory questions related to how participants typically prepared for a meeting with their healthcare provider and identified issues they might normally discuss or normally not discuss but wished they could. In provider groups, participants were similarly asked more exploratory questions about what they would like to know about their patients with MCC as well as how these assessments could be integrated into busy primary care practices. These questions were aimed at inductively identifying content from the perspective of participants (i.e., content elicitation). Sample questions asked at each stage of the focus groups are indicated in Table 1.

**Table 1:** Focus group questions by stage of PETS-Now development

Stage	Sample questions
Content elicitation (i.e., exploring new topics) and PRO workflow	• Patient focus groups o Consider the information you currently provide directly to your primary care team during a typical appointment. Imagine you are getting ready for a conversation with your clinical team, and you are making a list of topics you want to cover. What are the general topics that are typically most important for you to discuss with them? o Would you be willing to answer the questions at home, say a day or two before a scheduled appointment? How long would you be willing to spend answering the questions? • Provider focus groups o Consider topics that you would like to discuss with your patient but normally do not? These are things that you wish you could talk to them about, but that don’t ever seem to come up, or things that you are not sure how to ask about. What topics might be important?
Review of PETS content domains (i.e., confirming existing topics)	• Patient and provider focus groups o What content domains (i.e., themes) should definitely be included or definitely be dropped from the electronic tool? Which content domains are on the border? o Are there some content domains that can be combined, i.e., they are similar enough to be expressed as a single domain on the tool?
Prototype testing for usability	• Patient focus groups o What are your impressions of how easy or hard the tool is to use? How much help do you think patients will need learning how to use it? o What do you think of the overall look of it (including the colors and size of the text)? o How easy or hard was it to figure out how to click on things or move between screens? • Provider focus groups o Are these the sorts of things you would like to know about your patients? Is there any other information collected that you would like to have reported back and available to you during the appointment with a patient? o How long do you think it would take to review a report like this with a patient? How well do you think this will fit into your workflow?

In both groups, patients and providers were then shown a list of topics representing all PETS content domains (i.e., scales) and asked to confirm the relevance of these topics as things they might want to discuss with their care team in a clinical encounter. Topics generated from exploratory, content-elicitation questions and topics endorsed in the confirmatory question sets were combined and participants completed a voting exercise (i.e., using stickers to vote for their top-rated topics among all topics on displayed lists) to narrow down those to be recommended for inclusion in the PETS-Now tool. Participants also gave general feedback on the length and workflow for obtaining information via an electronic patient-reported outcome (ePRO) tool.

Focus groups were audio-recorded, and a member of the study team attended the groups and wrote observational notes for analysis. Audio files and notes were reviewed and summarized in a top-line report (i.e., rapid and concise summary of the responses to key questions asked of participants), for example, summaries of topics typically discussed or not discussed in primary care visits and narrative summaries of participant impressions of the technology used to collect, display and track PROM responses [25]. Reports also included the results of voting exercises. Data were initially organized by stakeholder group (patients vs. providers). Analysis involved circulation of top-line reports to individuals on the full study team for comment, followed by a group meeting of team members to discuss findings and finalize recommendations. The study team was comprised of a multidisciplinary group of researchers and clinical investigators representing primary care and family medicine, nursing, health services research, and qualitative research.

Final recommendations for PETS-Now content and functionality were transmitted to a vendor (InputHealth, Vancouver, British Columbia, Canada) who developed a prototype of the PETS-Now tool that could be administered on a tablet with results viewable on a web interface. Patient and provider stakeholders were invited back for usability testing focus groups between April 30, 2018 and June 20, 2018 (Step 2). Focus group participants were asked to complete PETS-Now on the tablet, and then they were asked for input on topic coverage, functionality, and overall experience with the tool (see Table 1). The study team also presented mock-ups of reports that could be generated at the point of care and solicited input from patients and clinicians about content, format, and integration into clinical conversations.

Analyses were similar to those used in Step 1; however, in Step 2 the primary focus was on categorizing patient input into key usability domains. Data were summarized by group and reviewed by the study team. InputHealth revised the prototype based on stakeholder feedback and the study team performed additional testing of the functionality of the web-based app to ensure the stakeholder feedback had been addressed prior to finalizing it for the pilot test of acceptability.

### Phase 2: acceptability of using PETS-Now in routine clinical practice

The aim of Phase 2 was to evaluate acceptability of using PETS-Now in routine primary care clinical encounters. Our goal was to recruit a diverse group of 5–8 primary care providers at each site (including physicians, nurses, pharmacists, dieticians, and mental health practitioners) who were willing to use PETS-Now with patients (excluding Phase 1 provider participants). We used a quasi-experimental design whereby clinical care experiences for each recruited provider were first assessed in a set of patients under standard care conditions (i.e., no tool use) followed by an assessment of care experiences that integrated use of the PETS-Now tool with a separate set of patients. For the standard care condition, we looked to enroll 7–10 patients with MCC per provider, with an overall goal of 100 patients. Phase 1 patient participants were not actively excluded from Phase 2 as it was deemed highly unlikely that they would be recruited given the large size of the eligible patient pool compared to the small number of Phase 1 participants. A study coordinator approached patients when they arrived for a scheduled appointment. The coordinator explained the study prior to the appointment, completed informed consent, and instructed the patient to complete a pre-encounter survey of socio-demographic information. After the appointment, the coordinator asked both the provider and patient to complete a brief post-encounter survey that measured the quality of chronic illness care, overall care quality, and the extent of patient-centered interaction including whether the most important patient concerns were addressed.

Upon reaching the enrollment target for the standard care condition, each provider was switched to the PETS-Now implementation. This included recruiting a new group of 7–10 patients per provider with an overall goal of 100. The study coordinator again approached patients arriving for a scheduled appointment, explained the study, completed informed consent, and instructed them to complete the pre-encounter, socio-demographic survey. The study coordinator then gave the patient an iPad featuring the PETS-Now app and instructed them to follow the prompts, complete the questions, and then return the iPad to the coordinator. The study coordinator switched the iPad from a patient-facing data collection mode to a provider-facing report mode and pulled up a summary report of the patient’s responses. The iPad displaying the summary report was given to the provider when he/she entered the exam room. Some providers expressed a preference for receiving a paper version of the patient’s responses, in which case the study coordinator printed out the report and handed it to the provider before entering the exam room. All providers received brief training on how to navigate the report prior to using the tool.

After the appointment, patients and providers were asked to complete the same post-encounter surveys that were used in the standard care condition. Additionally, both groups completed several questions about the PETS-Now tool. For patients, this included the following: (a) ease of use, (b) focus on biggest concern, (c) time to review results report, (d) comfort with discussing results report, and (e) willingness to use PETS-Now at a future visit. Providers answered the same queries and some additional questions about whether they went over the PETS-Now report with the patient and whether they learned anything new about the patient’s experience with treatment or self-management. After completing the accrual target, each provider was also given the opportunity to provide general impressions of the PETS-Now tool via a study closure survey.

We defined evidence of acceptability as at least 75% of patients reporting the iPad as being “somewhat easy” or “very easy” and at least 75% of patients reporting that they are “quite willing’ or “very willing” to use the PETS-Now tool again. When the sample size is 100 patients in the PETS-Now group, we can report a proportion of 0.75 with a 95% confidence interval of +/- 0.085. The sample size of 100 patients per group also supported the main secondary efficacy outcome of communication quality. For a two-sample t-test with alpha = 0.05 and power = 80%, we can detect an effect size of 0.4 or larger in mean communication scores between the two groups with equal sample size of 100 each. The acceptability questions were written by the study team, and the communication quality scale was modified from the Consumer Assessment of Healthcare Providers and Systems (CAHPS) Clinician and Group survey [26].

### Covid-19 modifications

While development of the PETS-Now tool was completed in 2018, the Covid-19 pandemic impacted the Phase 2 study protocol. Recruitment to the study was temporarily paused in March 2020 to allow both sites to cope clinically with the impact of the crisis on patient care. At the time of this accrual shutdown, several providers had already completed accrual of patients to the standard care condition and had switched over to the intervention of using the PETS-Now tool with patients. Prior to the pause, 27 patients (17 Mayo Clinic, 10 Hennepin Healthcare) had used the PETS-Now tool on an iPad during an appointment with a healthcare provider in the manner stipulated by the study protocol. Following the pause, the Hennepin Healthcare site re-opened to accrual in June 2020, while the Mayo Clinic site resumed accrual in July 2020. The clinics at both sites resumed patient appointments via telemedicine, so the study team modified the protocol to enable remote recruitment and administration of study materials. This included the following modifications applicable to both study conditions: (1) mailing a pre-visit letter to patients describing the study, (2) phoning patients up to 7 days before a scheduled appointment to further explain the study, answer questions, consent the patient, and complete the pre-encounter survey with the study coordinator, and (3) phoning patients to complete the post-encounter survey with the study coordinator 1 to 7 days after the appointment with the provider. As in the original protocol, provider participants completed the post-encounter survey after the appointment and returned it to the study coordinator.

For patients accrued to the intervention condition during the pandemic, the PETS-Now questions were administered over the phone by the study coordinator during the pre-encounter phone call. The coordinator entered responses into the iPad, generated the summary report of results, and emailed the report to the provider prior to the patient’s appointment. The Hennepin site adhered to this modified protocol for the remainder of the study, whereas the Mayo site reverted back to some in-person PETS-Now administrations when the clinics returned to in-person visits.

### Data analyses of pilot study

Analyses are mostly descriptive (e.g., frequencies of survey items). Chi-square analyses and t-tests are used to explore differences in responses between the standard care (control) and PETS-Now (intervention) conditions (α = 0.05).

## Results

### Phase 1: development of PETS-Now

**Step 1 – Content confirmation** Two focus groups were conducted with 15 patients with MCC (8 at Mayo Clinic and 7 at Hennepin Healthcare) and two focus groups were conducted with 18 healthcare providers (10 at Mayo Clinic and 8 at Hennepin Healthcare). Overall, patients were mostly non-Hispanic White (80%) and female (60%). Greater racial diversity was observed among patients at Hennepin Healthcare (43% African American). Average age of all focus group patients was 70 years and they self-reported an average of 4 chronic health conditions. A diverse range of providers was represented in the provider groups including 8 physicians, 6 nurses, a physician assistant, a social worker, and a community health worker.

Summary results of the concept elicitation exercise are shown in Table 2, grouped and displayed by participant type. Topics that were mentioned by participants in all groups include those related to monitoring health and health changes, advocating for oneself and determining priorities for health and life, social support and social roles, medications, as well as diet, sleep, and exercise. Regarding the review of PETS content, focus group participants endorsed all existing PETS thematic content domains for inclusion in the point-of-care tool. They also endorsed inclusion of all items within each content domain (i.e., all items within each domain scale). Sleep quality was an additional aspect that was not previously included in the PETS measure but was nominated and endorsed by both patients and providers in the content elicitation exercise. Sleep quality was added to the domain termed “Living Healthy,” which also included questions about diet and exercise. (Note, based on patient and provider feedback, some of the original domains of the PETS questionnaire were combined to reduce the number of content areas that the patient would have to consider, e.g., diet, exercise, and sleep were combined into a “Living Healthy” domain.) Results of the voting exercise to assess perceived importance of topics covered by the PETS as well as those emerging in the content elicitation exercise can be found in Additional file 1. Patients stressed that the resulting tool would need to be completed in 5 to 7 min. They reported a preference to be able to select more than one domain of treatment burden to discuss with the provider. Conversely, providers directed the study team to limit the choice of domains for consideration to just one as they were concerned that having to address more would lengthen the encounter.

**Table 2:** Top-line report of topics that patients might bring to a provider visit (concept elicitation)

Patients	Providers
• Change in conditions/symptoms	• What’s going well or not well since last visit
• Understanding health/monitoring	• Understanding of diagnoses and treatments
• How am I doing?	• Prioritizing conditions
• Meeting targets/ monitoring numbers • Difficulty monitoring (breathing/dizzy)	--
• Learning about health and asking about new research/treatment for existing conditions	• Health education and information seeking
• Understanding health information	• Role as patient
• Taking care of self	• Support systems (getting and giving)
• Understanding from others (social settings and respecting your needs) • Caretaking for others	• Social support• • Social stress or stress reduction • Living situation • Roles and responsibilities
• Advocating for own health care • Genuine interest in patients	• What’s important/patient goals and perspective of “health” • Life and health goals (including barriers and mismatch) • Leisure activities
• Traveling and dietary restrictions • Traveling with family and family support	--
• Review of current history • Repeating medical history	• Preventive care schedule • History/health conditions
• Medications/new medications • Side effects/interactions • Planning/schedules • Over-the-counter medications • Adjusting/coordinating medications • Cost of medications • Number of medications • Prior authorizations/paperwork bureaucracy • Planning unpredictability	• Paying for medications
• Sleep problems • Diet, exercise and sleep • Worry/sleep problems	• Diet, exercise and sleep
• Health care since last visit, e.g. surgeries	• Following prescribed care (why/why not)?
• Fear of future • Angry • Scared	• Understanding physical, functional, and emotional well-being • How are you doing? • Coping w/conditions • Fear of future
--	• Understanding follow-up plans and visits • Understanding information from providers
--	• Housing and transportation

**Fig. 2:**
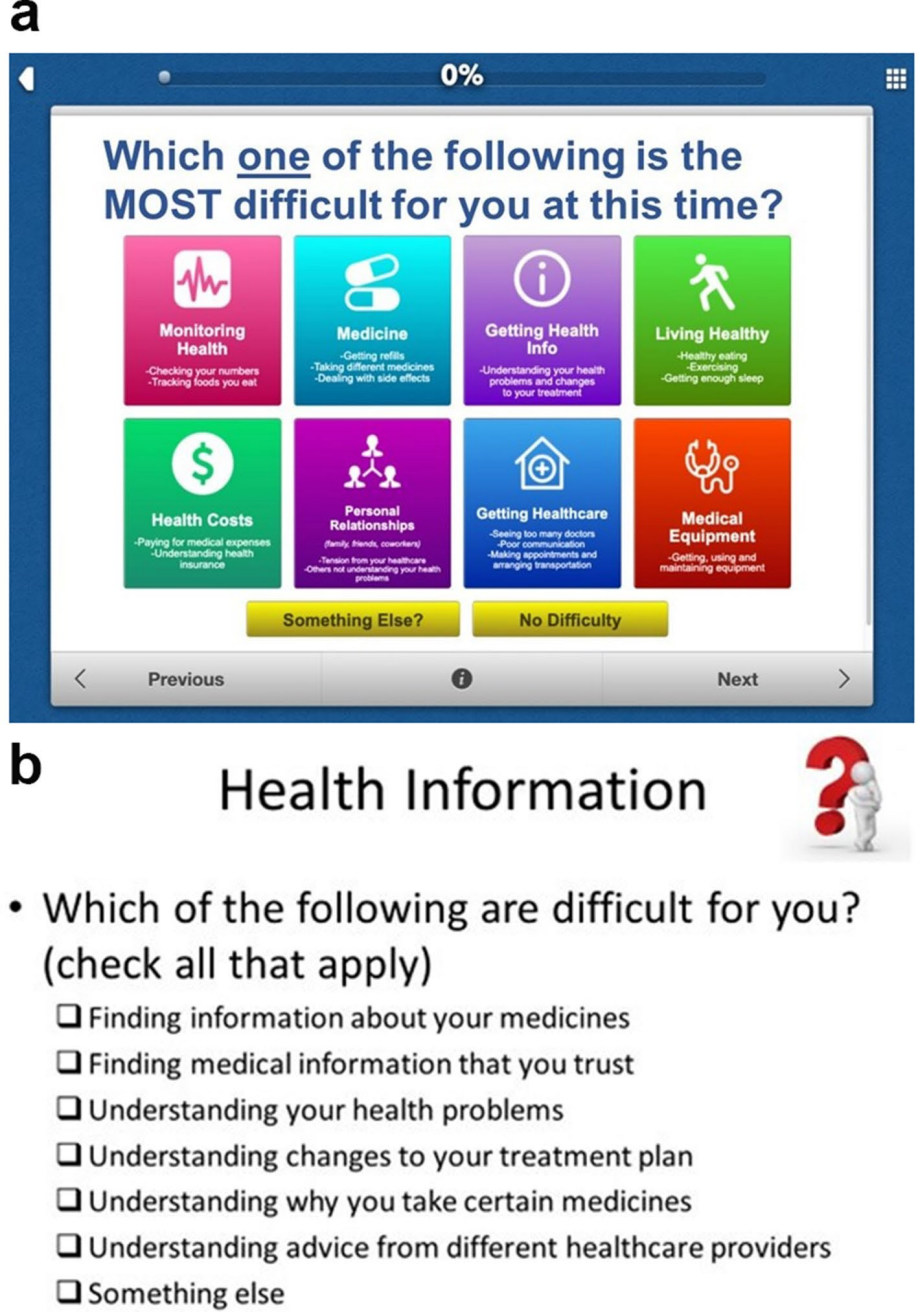
PETS-Now prototype screens: Main intake screen (**2a**) and Sample checklist (**2b**). PETS-Now, © 2020 Mayo Foundation for Medical Education and Research. All rights reserved

**Step 2 – Prototype testing for usability** Across both sites, nine patients and 13 providers attended a group to evaluate the usability of the PETS-Now prototype developed from the Step 1 results. The prototype featured a main screen displaying eight general domains of treatment burden (Fig. 2a). Upon reviewing the intake screen, the patient is instructed to select the one domain that is the most difficult for them at the current time. Alternatively, the patient can select “something else” if the most important concern was not listed or “no difficulty” if there are no concerns to report at the present time. As the focus of this work was on integration into busy clinic settings, we opted to limit the patient’s selection to a single burden domain to maximize the chances of provider uptake of the tool, but with broader categories based on patient feedback for greater relevance. Once the main domain is selected, the patient is then presented with a checklist of aspects of self-management germane to this domain and instructed to mark all that apply (see Fig. 2b for a sample). Checklists were adapted from the questionnaire items of the full PETS. After endorsing any relevant issues from the checklist, the patient is then presented with two groups of rating scale questions. The first group of questions assess the overall impact of self-management on well-being. This includes the two “impact” scales of the PETS questionnaire, the 6-item role-activity limitations scale and 5-item physical/mental exhaustion scale [17]. The second group of questions includes three items assessing overall quality of life, general physical health, and general mental health.

Providers offered feedback that anticipated what their older patients might experience when interacting with the tool. For example, several questioned whether patients would know how to scroll on an iPad to see additional items or whether they could read some of the smaller font. Patients struggled more than providers with touchscreen responsiveness, which was alleviated when they were provided with a stylus to use instead of their fingers. Both providers and patients felt the time to complete was reasonable (ranging from 5 to 10 min). Both groups requested better descriptions of the main concern categories on the intake screen and improvements to some instructions. Many patients and providers failed to notice the “Something else” and “No difficulty” buttons on the intake screen. A summary of the patient and provider comments that emerged from the prototype testing can be found in Additional file 2.

### Phase 2: pilot study to evaluate acceptability of PETS-Now

Seventeen healthcare providers (11 from Hennepin Healthcare, 6 from Mayo Clinic) agreed to recruit patients to the study. Average age of the group was 44.4 years and there were more women (11) than men. Five identified as racial-ethnic minorities (African American, Asian, or Other). Physicians were the predominant provider type represented (9); however, other providers included nurse practitioners (3), pharmacists (2), a physician assistant, a registered nurse, and a licensed psychologist. Recruitment to the study began in April 2019 and ended in August 2021. The previously described Covid-19 protocol modifications began in June 2020. Their impact on the results is described below.

Recruited providers saw 92 patients under a standard care (control) condition and another 90 patients under conditions in which the PETS-Now tool was implemented (intervention). Descriptive characteristics of patients in the two study conditions appear in Table 3. There were no significant differences between these groups on any sociodemographic characteristic (e.g., age, sex, race/ethnicity, education, marital and occupation status, living situation). Over 30% of patients in both study conditions represented a racial or ethnic minority (i.e., non-White). The groups were also similar on self-rated physical and mental health and indicators of health literacy. Notably, ≥ 40% of patients in both study groups rated their physical health as ‘fair’ or ‘poor’.

**Table 3:** Patient descriptive characteristics (acceptability study)

	Control (*N* = 92)	Intervention (*N* = 90)	p-value^1^
**Age in years**: Mean (std. dev.)	59.9 (15.4)	57.5 (15.8)	.24
**Female**: N (%)	49 (53%)	43 (48%)	.19
**Race / ethnicity**: N (%)			
Non-Hispanic, White	57 (62%)	60 (67%)	.26
Non-Hispanic, Black Hispanic, other or multiple races	27 (29%) 8 (9%)	18 (20%) 12 (13%)	
**Education level**: N (%)			
College-educated	67 (73%)	63 (70%)	.07
High school or less	25 (27%)	27 (30%)	
**Married or living with a partner**: N (%)	41 (45%)	35 (39%)	.90
**Current living situation**: N (%)			
Living in home or apartment	85 (92%)	79 (88%)	.11
Assisted living or nursing home	3 (3%)	9 (10%)	
Homeless	4 (4%)	2 (2%)	
**Physical health rating**: N (%)			
Excellent / very good	22 (24%)	20 (22%)	.99
Good	31 (34%)	33 (37%)	
Fair / poor	38 (41%)	35 (39%)	
Missing	1 (1%)	2 (2%)	
**Mental health rating**: N (%)			
Excellent / very good	38 (42%)	37 (42%)	.99
Good Fair / poor	27 (30%) 25 (28%)	26 (29%) 26 (29%)	
**Difficulty understanding written medical information**: N (%)			
Always / often	5 (5%)	5 (6%)	.87
Sometimes	13 (14%)	11 (12%)	
Occasionally / never Missing	72 (78%) 2 (2%)	73 (81%) 1 (1%)	
**Difficulty understanding medical information provided** **verbally**: N (%)			
Always / often	4 (4%)	6 (7%)	.90
Sometimes	15 (16%)	14 (16%)	
Occasionally / never	71 (77%)	70 (78%)	
Missing	2 (2%)		

Legend ^1^Comparison of control vs. intervention

Among the 90 participants in the intervention group, all eight of the PETS-Now domains were endorsed as an important current concern by at least two patients. Frequencies of patient endorsement were: Living Healthy (42%), Health Costs (9%), Monitoring Health (8%), Medicine (8%), Personal Relationships (7%), Getting Healthcare (6%), Getting Health Information (3%), and Medical Equipment (2%). Several patients (12%) endorsed ”No Difficulty” at the time of the appointment or “Something Else” (2%).

**Patient impressions of encounter (control vs. PETS-Now intervention)** Responses to questions about the care quality of the visit with the provider are shown in Table 4. There were no significant differences on any of the post-encounter questions between patients in the control and intervention groups. Overall care quality was assessed on a 0 (worst possible) to 10 (best possible) scale and was high in both study groups. The mean overall rating of the visit with the provider was 9.2 and 9.3, respectively for the intervention and control groups.

**Table 4:** Patient post-encounter survey responses

	Control (*N* = 91)^1^	Intervention (*N* = 84)^1^	p-value^2^
**Did healthcare provider consider how difficult or challenging it is for you to manage your chronic conditions in your daily life?** N (%)			
Very much / quite a bit	76 (84%)	71 (85%)	.77
Somewhat / a little bit	10 (11%)	8 (10%)	
Not at all	2 (2%)	3 (4%)	
Missing	3 (3%)	2 (2%)	
**Did healthcare provider give you suggestions of how to take care of your chronic conditions even in hard times?** N (%)			
Very much / quite a bit	71 (78%)	74 (88%)	.09
Somewhat / a little bit Not at all Missing	13 (14%) 1 (1%) 6 (7%)	6 (7%) 2 (2%) 2 (2%)	
**Did healthcare provider seem interested in how taking care of your chronic conditions affects your life?** N (%)			
Very much / quite a bit	82 (90%)	75 (89%)	.60
Somewhat / a little bit Not at all Missing	6 (7%) 0 (0%) 3 (3%)	5 (6%) 2 (2%) 2 (2%)	
**Did healthcare provider know the important information about your medical history?** N (%)			
Very much / quite a bit	82 (90%)	77 (92%)	.93
Somewhat / a little bit Not at all Missing	5 (5%) 0 (0%) 4 (4%)	5 (6%) 0 (0%) 2 (2%)	
**Did healthcare provider spend enough time with you?** N (%)			
Very much / quite a bit	83 (91%)	78 (93%)	.05
Somewhat / a little bit	5 (5%)	3 (4%)	
Not at all Missing	0 (0%) 3 (3%)	1 (1%) 2 (2%)	
**Mean rating of overall care**			
**quality? (0-worst; 10-best)**	9.3 (SD = 1.3)	9.2 (SD = 1.4)	.57

Legend ^1^One patient in the control condition and 6 patients in the intervention condition did not complete and return the post-encounter survey. ^2^Comparison of control vs. intervention

**Fig. 3:**
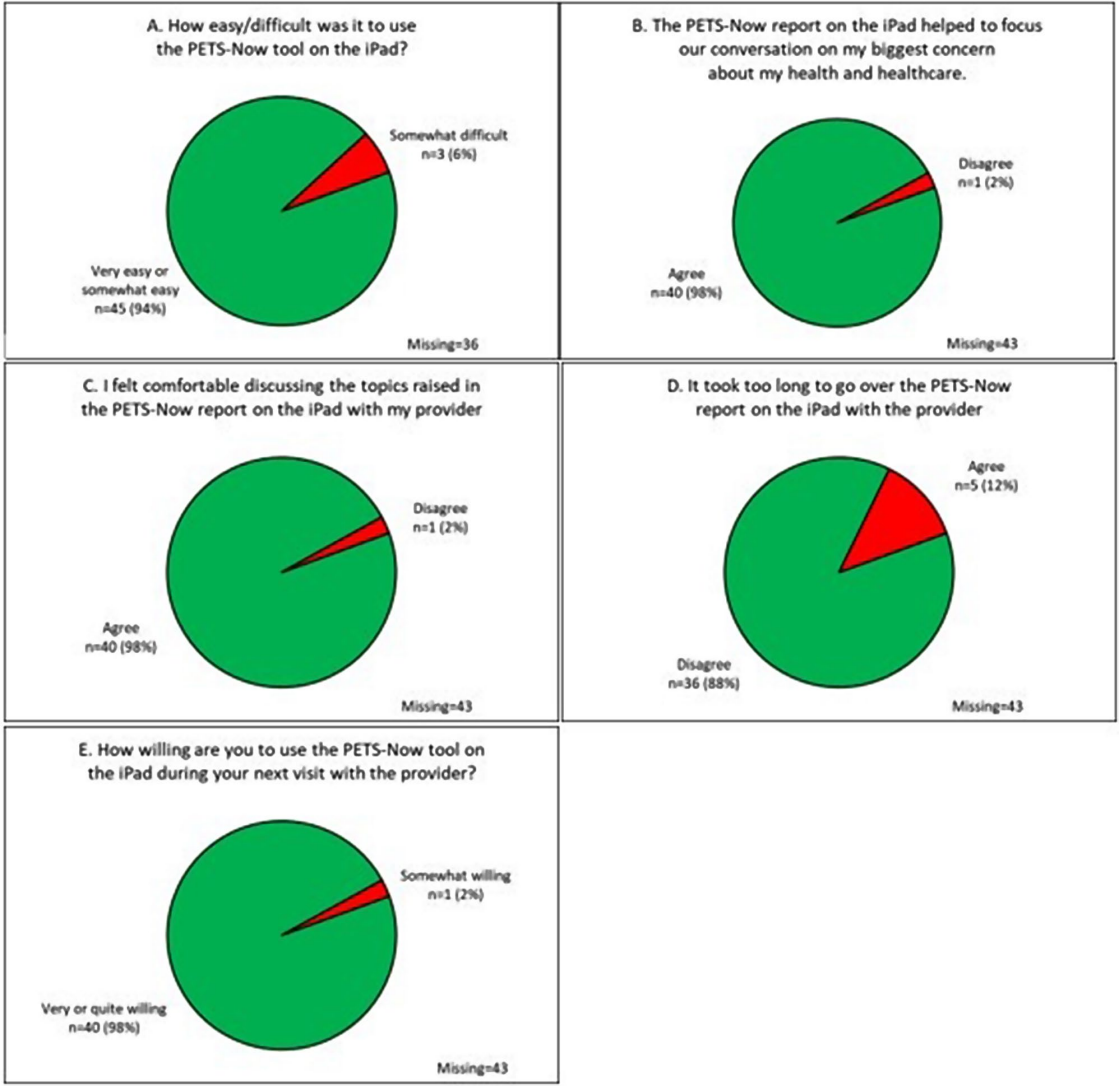
Patient ratings of the PETS-Now tool (intervention condition only: panels **A-E**). Legend: Responses reflect patients who self-administered the PETS-Now on the iPad tablet. Responses are missing for those patients who were administered the PETS-Now by research assistant interview during the Covid-19 pandemic because these questions were not asked of these patients. Percentages in each panel reflect the valid percent of those who self-administered the PETS-Now via the tablet

**Patient evaluation of PETS-Now** Fig. 3 displays patient responses to the questions evaluating the PETS-Now tool. The figure represents responses of those patients who self-administered the PETS-Now on the iPad tablet. Responses are missing for patients who were administered the PETS-Now over the phone by an interviewer during the Covid-19 pandemic as these questions were not asked of these patients. As shown in Fig. 3, the large majority of patients who self-administered the PETS-Now found it very or somewhat easy to use (94%), agreed that it helped to focus the conversation with the provider on their biggest concern (98%), and felt comfortable discussing the topics raised by the PETS-Now with the provider (98%). Most patients (88%) did not feel that the PETS-Now report took too long to go over with the provider and almost all patients (98%) expressed being very or quite willing to use the PETS-Now at a future visit with the provider.

**Provider impressions of encounter (control vs. PETS-Now intervention)** As with the patients, provider impressions of the encounters were highly favorable across the control and intervention conditions (no significant differences). In almost all of the visits, providers agreed that their time was well spent (98% in the intervention condition, 99% in the control condition). Furthermore, in 95% of the visits in both study conditions providers agreed that they were able to address the patient’s most important current concern.

**Fig. 4:**
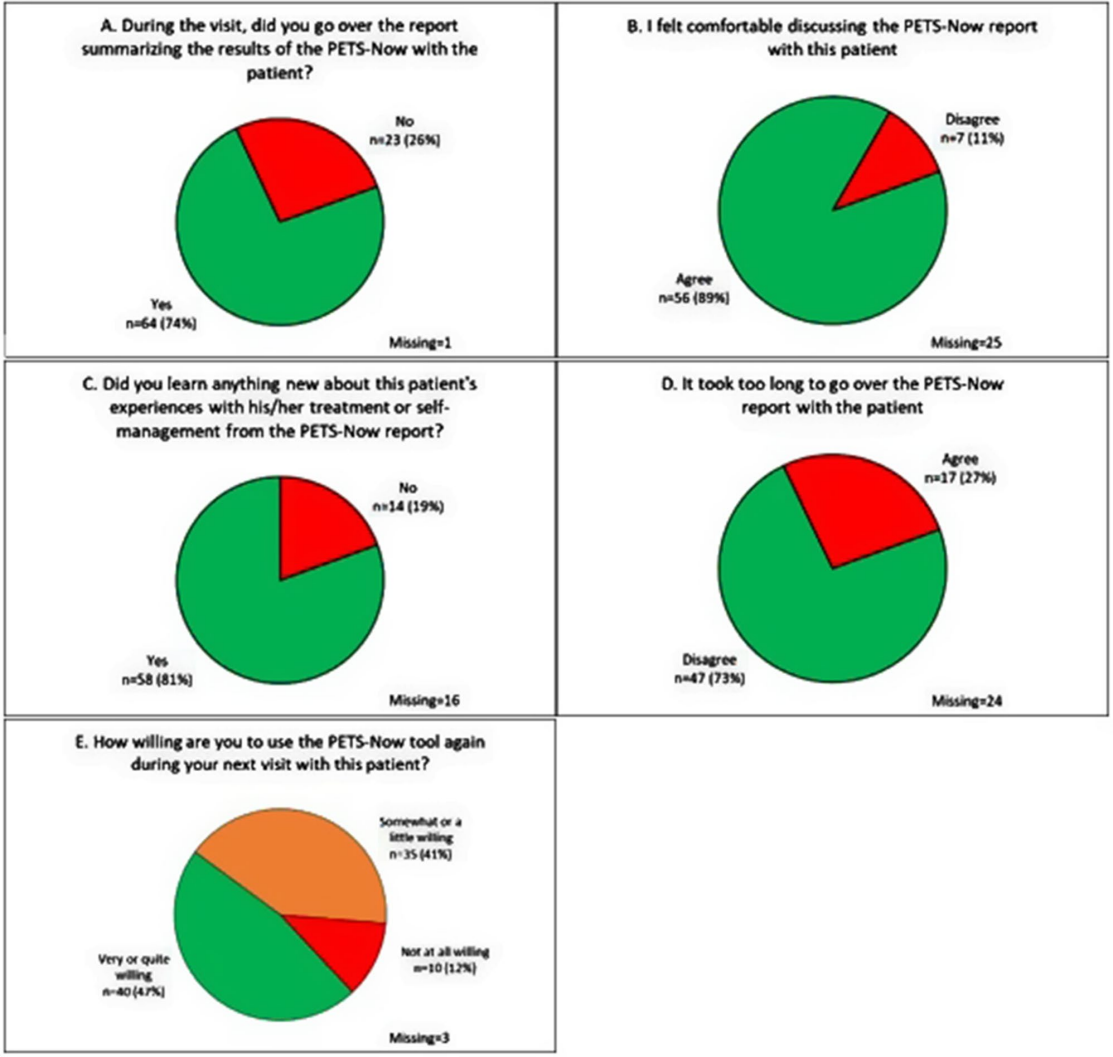
Healthcare provider post-encounter ratings of the PETS-Now tool (intervention condition only: panels **A-E**)

**Provider evaluation of PETS-Now** Fig. 4 displays provider responses to the questions evaluating the PETS-Now tool during each encounter in which it was used with a patient. In 74% of the encounters, providers reviewed the results of the PETS-Now with the patient. In most cases (89%), the providers felt comfortable discussing the PETS-Now report with the patient. Furthermore, they felt that they had learned something new about the patient’s experiences with his/her treatment or self-management in most encounters (81%). The providers did not feel that it took too long to go over the PETS-Now report in most of the visits (73%), and after 47% of the visits they reported being either ‘very’ or ‘quite’ willing to use the PETS-Now tool again at the next visit with the patient.

**Provider overall impressions of PETS-Now** At study closure, each provider was given the opportunity to provide overall impressions of the PETS-Now tool. While only 8 of the 17 providers completed this closure survey, opinions were generally favorable. Six of the 8 providers rated the PETS-Now as easy to use and five of them felt it would be ‘very’ or ‘quite’ feasible to integrate the tool into their visits with patients with MCC. Most of these providers (7 of 8) felt that the PETS-Now was effective at identifying opportunities to decrease treatment burden with patients through changing care plans or referral to support services. Six of the 8 providers reported that using the PETS-Now had changed their perspective on the challenges faced by patients in managing their health. Finally, providers were also given the opportunity to provide narrative impressions of the tool. Below is a sample of provider responses. This includes some noteworthy challenges such as lacking time to use the tool routinely in practice, the need to integrate the information into the electronic health record, as well as the need for guidance on how to use the information to address patient concerns.

Very helpful at identifying when patients are suffering challenges with their medications and treatment burden.

Doing this routinely would require a time investment. I don’t know if I could commit to using it routinely long term.

It is an excellent tool. I think our patients have burden in their care. I think it would be helpful to have this integrated into the EPIC medical record.

It would need to be integrated into the electronic health record to be useful.

[PETS-Now] Made me think more introspectively about the patient.

It made me aware of patients who were struggling, but it didn’t give me recommendations for how to address the patient’s concerns.

The information needs to be distilled and prioritized with actionable insights.

## Discussion

We developed an ePRO tool (the PETS-Now) to assess treatment burden in patients with MCC and share the information with primary healthcare providers to use during regular patient appointments. The tool leverages content from our previously validated measure of treatment burden (the PETS). Following development and usability testing in focus groups of patients and healthcare providers, we pilot tested the PETS-Now in clinic. Primary-care providers across two sites participated by first seeing a set of patients under usual clinic conditions (i.e., standard care), followed by encounters with a different set of patients whereby treatment burden information was collected using the PETS-Now pre-visit and made available to the provider for the patient appointment. The protocol required some modification during the study to accommodate alterations in clinic practices during the Covid-19 pandemic. Many clinic appointments occurred by telemedicine, requiring research assistants to administer the PETS-Now tool to the patient over the phone prior to the appointment and transmit the results report to the provider to have during the appointment.

While comparisons of patient ratings of care quality did not reveal any differences between the standard care and intervention conditions, in general, patients found that the PETS-Now was easy to use, were comfortable discussing the results with their provider, felt that PETS-Now helped focus the conversation with the provider on their biggest concern, and were willing to use the tool at future visits. Provider impressions of the patient encounters were also largely positive, regardless of whether the PETS-Now was used. Providers had many favorable impressions of the PETS-Now tool with most endorsing that they felt comfortable discussing the report with the patient and that they learned something new about the patient from the report. Moreover, most of the providers did not feel that it took too long to go over the report with the patient, and most expressed a willingness to use it again at the next appointment with the patient.

Results provide preliminary evidence of feasibility of using PETS-Now in clinic. Patients were able to complete the PROMs, and clinicians reported using the information in consultations. However, some providers suggested that usability of the PETS-Now could be enhanced by integrating the information collected into the patient’s electronic health record. Incorporating PRO data directly into the electronic health record can facilitate its review at the point of care as well as tracking it over time alongside other clinical information [27]. Integration of PROMs into clinical workflows will be essential to their sustained adoption and routine use. Providers also noted time concerns, stating that given time constraints they needed to limit the number of domains reviewed.

This study has several strengths. To our knowledge this marks the first attempt to develop and pilot test an ePRO tool designed specifically to assess issues of treatment burden and use that information to inform discussions with patients. PETS-Now is an adaptation of a previously validated measure of treatment burden that has been used with a range of patient populations [8, 17, 28–30]. Its content and formatting were vetted by primary healthcare providers and patients seen in primary care clinics, with racial and ethnic diversity represented in both stakeholder groups. A mix of provider types participated in both phases of the study including physicians, physician assistants, nurses, pharmacists, dieticians, and mental health practitioners. Integration of the tool into clinical practice was tested in two very different primary care settings: a private, multi-specialty integrated practice and a public safety-net hospital. Finally, the PETS-Now clinical tool augments other versions of the PETS designed for use in clinical research [31–33] and studies of quality of care [23].

Notwithstanding the strengths, there were some study limitations. First, the quasi-experimental design used was not the initial design choice for the pilot test. Randomization of providers to a standard care or intervention arm was considered impractical given the potential for contamination across providers working in the same clinic (i.e., unplanned interactions between providers about the content of the tool). Furthermore, a longitudinal design in which encounters with a patient would first occur under the normal standard of care followed by cross over to the use of PETS-Now at a later appointment was also deemed impractical given the possibility of missed or significantly delayed follow-up appointments. Second, rates of recruitment of patients per provider were variable. Rather than wait until the recruitment target for the standard care arm had been reached before implementing the PETS-Now intervention, the timing of the switch from standard care to intervention was done on a provider-by-provider basis. Third, the high patient ratings of care quality in both study conditions might indicate that the providers who agreed to participate were already highly patient-centered in their approach making it difficult to detect any differences between the conditions. Furthermore, possible ceiling effects of the care quality evaluation measures may have masked differences between study arms. Fourth, even after persistent efforts to follow-up with them, less than half of the providers who participated in the pilot test completed a study closure survey limiting the number of overall impressions we had from providers about the PETS-Now tool. No data were collected on reasons for this non-response. Fifth, interviewer phone administration of the PETS-Now, necessitated by the clinics’ switch to telehealth visits during the pandemic, meant that the tool could not be administered as it was originally intended via a touchscreen tablet at the point of care. This led to several questions being dropped from the post-encounter survey resulting in an incomplete patient evaluation of the PETS-Now. Finally, given that this study was conducted in the USA, we cannot say with certainty that all issues represented in the PETS-Now tool are equally relevant to patients residing in countries with different healthcare systems, e.g., those with government-subsidized healthcare.

### Comparison to other studies

Development and testing of interventions integrating valid PROMs to support primary care practice has occurred only recently. In the U.S., Monahan and colleagues [34] have developed SymTrak, a brief, clinically actionable self-report tool to routinely monitor symptoms common across a host of diseases and chronic conditions seen in primary care, especially among patients with MCC. This includes the prevalent and disabling “SPADE” symptom cluster (i.e., sleep disturbance, pain, anxiety, depression, and low energy/fatigue). SymTrak draws on the content of previously validated PROMs like the Patient-Reported Outcomes Measurement Information System (PROMIS), the Patient Health Questionnaire (PHQ), and Generalized Anxiety Disorder-7 (GAD-7), adapting them to facilitate efficient administration and usability. In feasibility testing, the tool appears to be acceptable to patients and caregivers, with most reporting that it was relevant, important, and easy to understand and complete [34]. Large-scale validation supported reliability, construct validity, and responsiveness to change of both a 23-item version and an abbreviated 8-item version [35, 36].

In the UK, Porter and colleagues [37] have developed and piloted a PROM intervention that provides real-time feedback and clinical management support to primary care nurses seeing patients with MCC. Patients complete standardized condition-specific PROMs (one per relevant condition), a generic PROM (the EQ-5D), and an individualized PROM (the Patient Generated Index) with results informing a nurse-led annual review of the patient’s health status. Among 68 patients and 12 nurses participating in the pilot, over 90% found that the information in the review was easy to understand and helpful at prioritizing the patient’s health issues, while over 80% found that the information was a helpful part of the annual review.

As in our study, these prior studies demonstrate that existing PROMs can be leveraged to build patient-centric tools that are feasible to integrate into clinical practice to inform primary-care teams about their multimorbid patients. However, while SymTrak and the nurse-oriented intervention of Porter and colleagues provide insight into a patient’s health status (i.e., symptoms and health-related quality of life), neither sheds light on patient treatment burden. Furthermore, unlike the PETS-Now, neither of these tools were designed up front to capture data electronically, although their developers note that future versions will enable this. Finally, efficacy testing of all these novel tools is still needed.

### Clinical implications

The PETS-Now provides patients with a means to inform their primary healthcare providers about the challenges they face in maintaining their treatment and self-care regimens. Since these issues may not spontaneously emerge during patient appointments, the tool can serve to prompt a conversation about treatment burden with the provider. Patients may find that the PETS-Now gives them permission to talk about issues that affect their ability to adhere to treatments and self-care, issues that they might not otherwise feel comfortable discussing with their provider [38]. As alluded to by providers participating in our study, there are important clinical considerations to bear in mind for subsequent tests of the tool. First, proper integration will require that the PETS-Now seamlessly fit into existing clinical pathways and routines, which can be expected to vary across clinics. Second, the tool should augment patient-provider interactions and never distract from or replace other important discussions about clinical concerns. In short, the burden tool should never become a burden to either patients or providers. Third, future iterations of the tool will need to specify clinical supports and/or actions that providers might consider in addressing any treatment burden concerns identified. Examples might include contacts with social work to address challenging social determinants of health, or referrals to pharmacy medication management for help with complex medication regimens [16]. Fourth, uploading patient responses directly into the electronic medical record may need to be prioritized to maximize clinician acceptance of PETS-Now. Finally, consideration should also be given to expanding the number of treatment burden domains that the patient is allowed to select. We limited selection to only a single domain to facilitate integration of the tool into the clinic, deferring to feedback from study providers who were concerned about the length of the encounter. However, a more comprehensive assessment of multiple domains might assist clinicians in tailoring care recommendations to the needs and situation of each individual patient. For older patients, this could be accomplished by using the PETS-Now at annual Medicare wellness visits where time is less of a limiting factor.

## Conclusions

The PETS-Now is a novel clinical tool adapted from a previously validated measure of treatment burden. It is intended to provide patients and healthcare providers with a platform to facilitate exchanges about treatment burden during regular clinic appointments. The PETS-Now was co-designed by patients and providers to enhance its acceptability. Patients found it easy to use and were comfortable discussing the topics it raised with their providers. Providers learned something new about their patients’ experience with treatment and self-management and seemed willing to use it again at future visits. Next steps could include a randomized clinical trial to determine the efficacy of using the PETS-Now tool in comparison to standard care. This would likely require cluster randomization by clinic site and perhaps a stepped-wedge design that involves sequential transition of clinics from standard care-control to use of the tool. Any future trial of PETS-Now will need to employ a robust set of outcomes, including measures of treatment burden, health-related quality of life, self-efficacy, patient satisfaction, and process indicators like patient-provider communication. Feasibility assessment (e.g., study accrual, protocol adherence, integration with existing clinical workflows) and qualitative inquiries to uncover barriers and facilitators of tool implementation will also be necessary.

## Electronic supplementary material

Below is the link to the electronic supplementary material.

**Additional file 1:** Content themes voting exercise (focus groups)

**Additional file 2:** Prototype testing comments from patients and providers

## Data Availability

The datasets generated and analyzed for this study are not publicly available as they are governed by a resource sharing plan of the funded project. De-identified datasets can be made available to interested investigators upon reasonable request and approval of the corresponding author and the institutions where the study was conducted, provided that all conditions of data sharing as stipulated in the resource sharing plan are met. All requests are subject to review by the project principal and co-investigators. All versions and adaptation of the PETS measure, including the PETS-Now, are protected by copyright, © 2020 Mayo Foundation for Medical Education and Research. All rights reserved.

## Abbreviations

ePRO: Electronic Patient-Reported Outcome
MCC: Multiple Chronic Conditions
PETS: Patient Experience with Treatment and Self-management
PROM: Patient-Reported Outcome Measure
IRB: Institutional Review Board
COPD: Chronic Obstructive Pulmonary Disease
PROMIS: Patient-Reported Outcomes Measurement Information System
PHQ: Patient Health Questionnaire
GAD-7: Generalized Anxiety Disorder-7
EQ-5D: EuroQol-5 Dimensions
