# Supplementary material for: Development and acceptability of PETS-Now, an electronic point-of-care tool to monitor treatment burden in patients with multiple chronic conditions: a multi-method study
Source: BMC Prim Care. 2024 Mar 1;25:77. doi: 10.1186/s12875-024-02316-5 (PMC10908048; doi:10.1186/s12875-024-02316-5)
Supplement: Supplementary file 1 — Additional file 1: Content themes voting exercise (focus groups) [file 12875_2024_2316_MOESM1_ESM.pdf]

Additional file 1. Themes sorted by total importance votes of all focus group participants

Theme	Total voting theme is "most important"	Total voting theme is "important"
Learning about health conditions/understanding diagnoses, medication dosing	7	8
Taking medications	7	5
Monitoring health status	5	8
Interpersonal challenges (social stressors, sex life, sexual function)	4	12
Health behaviors such as diet and exercise	2	13
Barriers to self-care including financial	2	10
Role or social activity limitations	2	6
Medical and health care expenses	1	10
Difficulty navigating healthcare systems (including expectations)	1	8
Medications/side or no effects/interactions/planning/schedules	1	4
Changes in condition/symptoms (Understanding them / Managing them)	1	3
Physical or mental exhaustion	0	12
Patient expectations related to care	0	5
Confusion about medical information	0	4
Financial impact (different than challenge)	0	4
Difficulty with healthcare providers including trust in western medicine and gender identity	0	3
Understanding health/monitoring	0	2
Medical equipment and devices	0	2
Sleep	0	2
Social determinants of health	0	2
Medical appointments	0	1
Advocating for self	0	1
Safety in home	0	1
Teamwork among providers	0	1
Navigating finance and insurance (task)	0	1
Mental health and chemical dependency	0	1
