# Supplementary material for: Development and acceptability of PETS-Now, an electronic point-of-care tool to monitor treatment burden in patients with multiple chronic conditions: a multi-method study
Source: BMC Prim Care. 2024 Mar 1;25:77. doi: 10.1186/s12875-024-02316-5 (PMC10908048; doi:10.1186/s12875-024-02316-5)
Supplement: Supplementary file 2 — Additional file 2: Prototype testing comments from patients and providers [file 12875_2024_2316_MOESM2_ESM.pdf]

Additional file 2: Prototype testing comments (patients and providers)

Screen	Issue	Requested Changes
Welcome	Acronym PETS made them think of companion animals.	• Spell out the acronym the first time used, as follows: Patient Experiences with Treatment and Self-management (PETS)<br>• Delete "PETS-Now"
Main concerns	Domains do not reflect "health issues", rather they reflect burden of treatment	• Change page title to read "Which the following is the MOST difficult for you at this time?"
Main concerns	Usefulness of icons was mixed. Favorable at Hennepin, negative at Mayo. Both groups wanted more text descriptions.	• Reduce size of icon to make room for a few more examples and larger font.<br>• Change health cost icon to a dollar sign $
Main concerns	Getting health information and getting healthcare icons are switched	Align icon with the correct domain.
Main concerns	White text on light colored boxes was hard to read	Possible solutions<br>(1) Use bold colors to outline the boxes. Text inside can be black on a white background. Icon color inside the box can match that of border.<br>OR<br>(2) Explore other designs for better readability while maintaining intuitive design<br><<Explore solutions with tech. vendor>>
Main concerns	Domain labels: Vendor changed a few labels/examples for brevity. Some labels and examples didn't work well with patients	• Add parenthetical back to "Personal Relationships", e.g., "Personal Relationships (family, friends, coworkers)"<br>• Reword "tension from your healthcare" to "Healthcare causing tension with others."<br>• Add more examples from the Oct 2017 mock up as space allows.<br>• Add "For example:" after domain name and before examples to make it more clear to patients that these are partial and not all inclusive lists.
Main concerns	Some patients and providers missed the "Something else" and "No difficulty"	• Make buttons more noticeable.
Getting health Care	Typo 2nd to last bullet	• Change "communication" to "communicate"

**Screen**	**Issue**	**Requested Changes**
Domain-specific drill down screens	Redundant/unnecessary/unclear instructions	• Delete "Optional: skip this if it does not apply to you."<br>• Change instructions to "Check all that apply. If none apply, scroll up to select a different issue."
Interference questions	Not obvious that there are more questions "below the fold." Patients answered first question and clicked "next," which brought up the warning message.	(1) [Preferred if possible] Keep question context/instructions frozen at top of screen (reducing font a bit is OK). Auto scroll to next question after person answers each question. Similar to how autoscroll is used with xylophone buttons. Allow manual scrolling back up to change previous questions in scale if desired.<br>OR<br>(2) Provide a visual cue immediately upon launching this screen to inform patients that more questions are below the fold and that they need to scroll to see them.<br>OR<br>(3) Shrink the size of the instructions and questions so that more than 1 question is visible on an iPad screen with the initial launch of this screen.
Interference, Exhaustion, well-being	Warning message. Patients didn't know what to do to make the warning message go away. Most tried to 'Move to the question' by tapping outside of the warning box.	Change message to "Click here to move to the next question"
Interference, Exhaustion	Instructions were forgotten as patient moved through the scale.	Repeat instructions in middle of scale – after 3rd question.
Exhaustion	Many patients admitted they didn't read the instructions. They thought these questions were about their feelings in general and not due to the work of managing their health.	Underline "tasks and activities" in the instructions to call attention to the qualifier. Do this also for Interference questions for consistency.
Exhaustion	Some found "preoccupied" difficult to understand.	No change – need to maintain fidelity to the original paper survey to allow comparison
End screen	Many thought "Go Home" was poor wording.	Replace with "End"
